# Two chromosomal reference genome sequences for the malaria mosquito, *Anopheles *( *Nyssorhynchus*) *darlingi*, Root, 1926 from French Guiana and Peru

**DOI:** 10.12688/wellcomeopenres.23989.1

**Published:** 2025-04-10

**Authors:** Mathilde Gendrin, Katy Heu, Marta Moreno, Dionicia Gamboa, Joseph M Vinetz, Carlos Tong, Jan E Conn, Harriet F Johnson, Haynes Heaton, Martin G Wagah, Joanna C Collins, Ksenia Krasheninnikova, Sarah E Pelan, Damon-Lee B Pointon, James W Torrance, Alan Tracey, Marcela Uliano-Silva, Jonathan M D Wood, Katharina von Wyschetzki, Wellcome Sanger Institute Scientific Operations: Sequencing Operations, Shane A McCarthy, Mara K N Lawniczak, Daniel E Neafsey, Alex Makunin

**Affiliations:** 1 Microbiota of Insect Vectors Group, Institut Pasteur de la Guyane, Cayenne, Cayenne, French Guiana; 2 Department of Infection Biology, London School Of Hygiene and Tropical Medicine, London, England, UK; 3 Laboratorio de Malaria: Parásitos y vectores, Laboratorios de Investigación y Desarrollo, Facultad de Ciencias e Ingeniería, Universidad Peruana Cayetano Heredia, Lima District, Lima Region, Peru; 4 Section of Infectious Disease, Department of Internal Medicine, School of Medicine, Yale University, New Haven, Connecticut, USA; 5 GERESA Loreto Gerencia Regional de Salud Loreto, Unidad de Entomología, Iquitos, Peru; 6 Wadsworth Center, New York State Department of Health, Albany, NY, USA; 7 Department of Biomedical Sciences, College of Integrated Health Sciences, SUNY The State University of New York, Albany, New York, USA; 8 Scientific Operations, Wellcome Sanger Institute, Hinxton, England, UK; 9 CSSE, Auburn University, Auburn, Alabama, USA; 10 Tree of Life, Wellcome Sanger Institute, Hinxton, England, UK; 11 University of Cambridge Department of Genetics, Cambridge, England, UK; 12 Department of Immunology and Infectious Diseases, Harvard T.H. Chan School of Public Health, Boston, MA, USA; 13 Infectious Disease and Microbiome Program, Broad Institute, Cambridge, Massachusetts, USA

**Keywords:** Anopheles darlingi, South American malaria vector, genome sequence, chromosomal

## Abstract

We present two genome assemblies, each generated from individual female *Anopheles* ( *Nyssorhynchus*) *darlingi* (the malaria mosquito; Arthropoda; Insecta; Diptera; Culicidae), from wild populations in French Guiana and Peru. The genome sequences are approximately 180 megabases in span. The majority of each assembly is scaffolded into three chromosomal pseudomolecules with the X sex chromosome assembled. The complete mitochondrial genomes were also assembled and are both 15.4 kilobases in length. The assemblies differ by two inversions in chromosome arm 2R.

## Species taxonomy

Animalia; Arthropoda; Insecta; Diptera; Culicidae; Anophelinae; Anopheles; *Anopheles darlingi*; Root, 1926 (NCBI txid:43151).

## Background

The *Anopheles darlingi* mosquito is the most important Neotropical malaria vector, spanning an enormous geographic range from southern Mexico to northern Argentina ^1, 2^. It belongs to a subgenus ( *Nyssorhynchus*) that exhibits considerable evolutionary divergence from other anopheline subgenera ^3^ harbouring familiar malaria vectors from Africa and Asia, resulting in the proposal to recognize *Nyssorhynchus* as a genus-level classification ^4^. Given the importance of *An. darlingi* as a neotropical vector, the first short-read genome assembly was published in 2013, based on a collection of male and female mosquitoes from the Brazilian Amazon ^5^. The resulting assembly provided a first understanding of this divergent lineage of mosquitoes, but was highly fragmented (over 8000 scaffolds, N50 scaffold length = 81 kb) due to heterozygosity in the sequencing template from many individual mosquitoes and the inherent difficulty of assembling highly contiguous genomes from short reads.

Consequently, improved genome assemblies of *An. darlingi* were produced as part of the Anopheles Reference Genomes Project (PRJEB51690). Here we present two chromosomally complete genome sequences for *Anopheles darlingi*, based on single female specimens from Macouria, French Guiana and Cahuide, Loreto Department, Peru. The latter is derived from the first sustained colony of *Anopheles* ( *Nyssorhynchus*) *darlingi* established in 2013 from 135 wild-caught female mosquitoes obtained in the northeastern Peruvian Amazon region of Iquitos in the Loreto Department ^6^, for which maintenance of heterozygosity was demonstrated. This colony had no significant evidence of a bottleneck, decrease in total alleles, or increase in inbreeding compared with field specimens (founder population). Low-moderate differentiation between field and laboratory populations was detected ^7^. The genome assembly for the specimen from French Guiana has been annotated by NCBI RefSeq to support its use as an updated reference for the species. 

## Genome sequence report for specimen from French Guiana

The genome was sequenced from a single female *Anopheles darlingi* collected from Macouria, French Guiana (5.015, –52.528). A total of 32-fold coverage in Pacific Biosciences single-molecule HiFi long reads (N50 11.387 kb) were generated. Primary assembly contigs were scaffolded with chromosome conformation Hi-C data from a female sibling. Manual assembly curation corrected 13 missing joins or misjoins, reducing the scaffold number by 5.8%.

**Figure 1.**
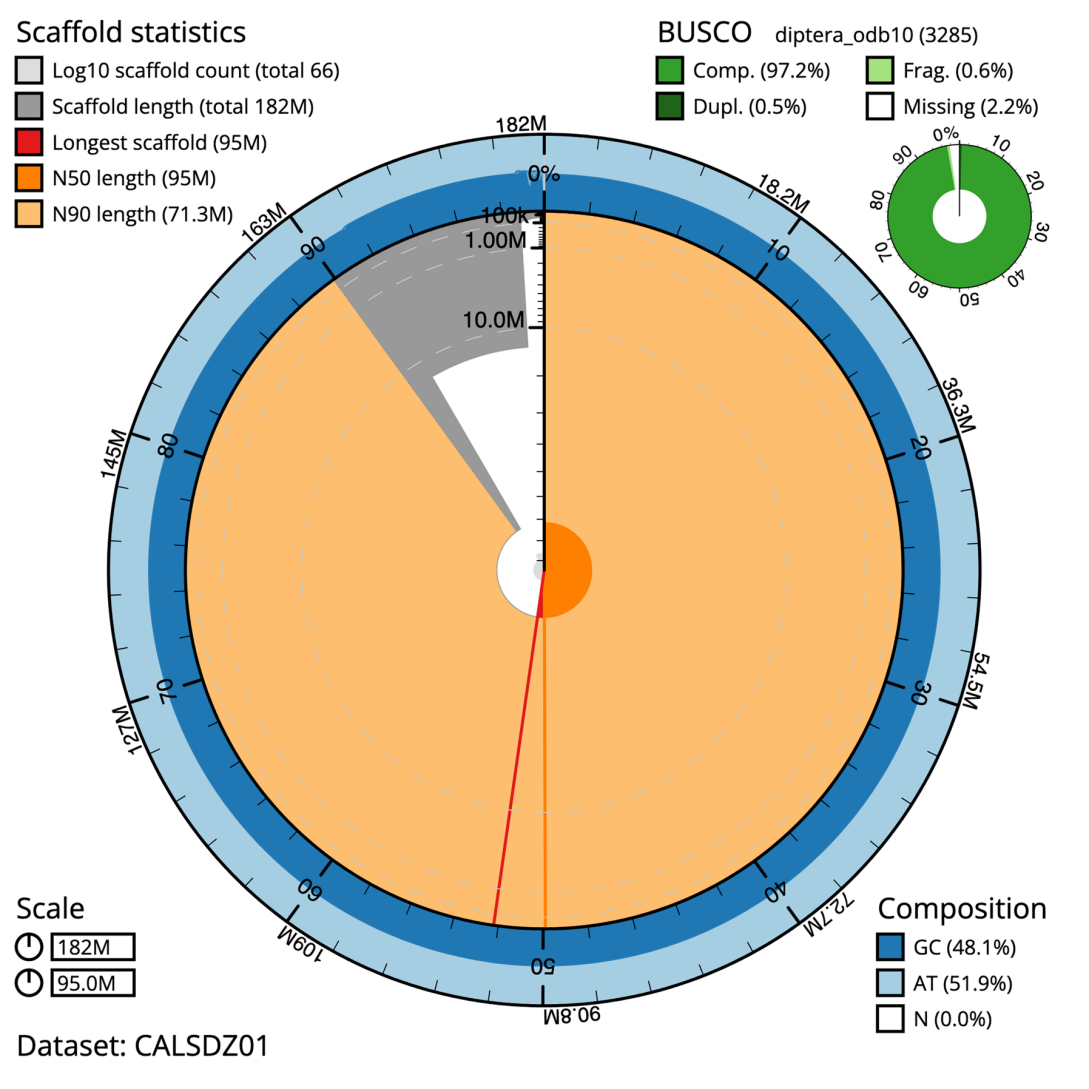
Snail plot summary of assembly statistics for *Anopheles darlingi* assembly idAnoDarlMG_H_01. The main plot is divided into 1,000 size-ordered bins around the circumference with each bin representing 0.1% of the 181,652,731 bp assembly. The distribution of sequence lengths is shown in dark grey with the plot radius scaled to the longest sequence present in the assembly (94,951,917 bp, shown in red). Orange and pale-orange arcs show the N50 and N90 sequence lengths (94,951,917 and 71,270,736 bp), respectively. The pale grey spiral shows the cumulative sequence count on a log scale with white scale lines showing successive orders of magnitude. The blue and pale-blue area around the outside of the plot shows the distribution of GC, AT and N percentages in the same bins as the inner plot. A summary of complete, fragmented, duplicated and missing BUSCO genes in the diptera_odb10 set is shown in the top right. An interactive version of this figure is available at https://blobtoolkit.genomehubs.org/view/idAnoDarlMG_H_01/dataset/CALSDZ01/snail.

The final assembly has a total length of 182 Mb in 66 sequence scaffolds with a scaffold N50 of 94.952 Mb ( Table 1). 98.89% of the assembly sequence was assigned to three chromosomal-level scaffolds, representing two autosomes (numbered and oriented against the *An. albimanus* STECLA strain assembly ( 8; GCA_013758885.1)), and the X sex chromosome ( Figure 1– Figure 4; Table 2).

**Table 1.** Genome data for *Anopheles darlingi* from French Guiana, idAnoDarlMG_H_01.

*Project accession data*	
Assembly identifier	idAnoDarlMG_H_01
Species	*Anopheles darlingi*
Specimen	idAnoDarlMG-H_01
NCBI taxonomy ID	43151
BioProject	PRJEB53253
BioSample ID	ERS10527351
Isolate information	female, whole organism
*Raw data accessions*	
PacificBiosciences SEQUEL II	ERR9439499
Hi-C Illumina	ERR9356785, ERR9356786
PolyA RNA-Seq Illumina	ERR9356791, ERR9356792, ERR9356793
*Genome assembly*	
Assembly accession	GCA_943734745
Accession of alternate haplotype	GCA_943734775
Span (Mb)	181.653
Number of contigs	96
Contig N50 length (Mb)	19.203
Number of scaffolds	66
Scaffold N50 length (Mb)	94.952
Longest scaffold (Mb)	94.952
BUSCO * genome score	C:97.2%[S:96.7%,D:0.5%],F:0.6%,M:2.2%,n:3285

* BUSCO scores based on the diptera_odb10 BUSCO set using BUSCO 5.3.2. C=complete [S=single copy, D=duplicated], F=fragmented, M=missing, n=number of orthologues in comparison. A full set of BUSCO scores is available at https://blobtoolkit.genomehubs.org/view/idAnoDarlMG_H_01/dataset/CALSDZ01/busco.

**Figure 2.**
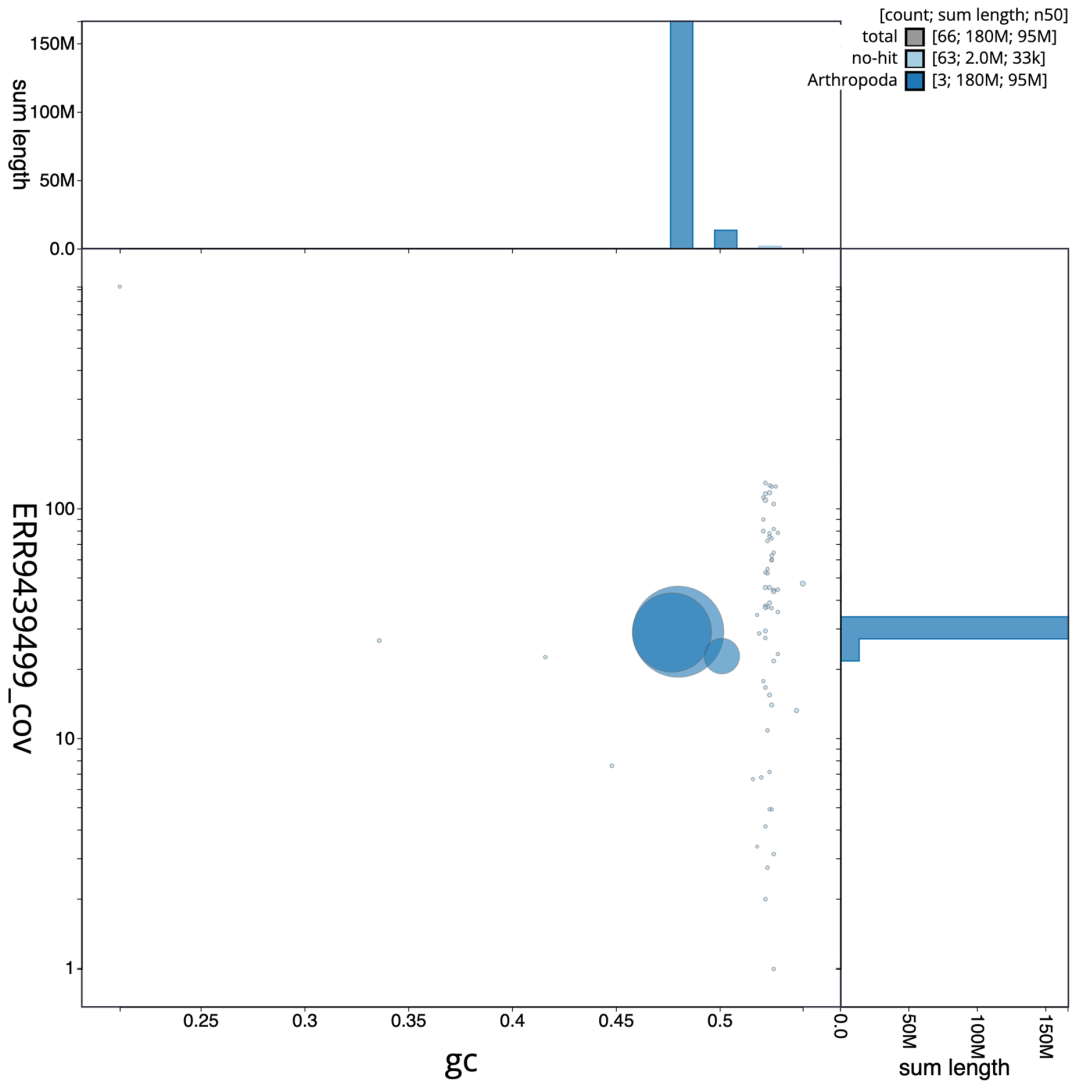
Blob plot of base coverage in idAnoDarlMG_H_01 PacBio HiFi reads against GC proportion for *An. darlingi* assembly idAnoDarlMG_H_01. Chromosomes are coloured by phylum. Circles are sized in proportion to chromosome length. Histograms show the distribution of chromosome length sum along each axis. An interactive version of this figure is available at https://blobtoolkit.genomehubs.org/view/idAnoDarlMG_H_01/dataset/CALSDZ01/blob.

**Figure 3.**
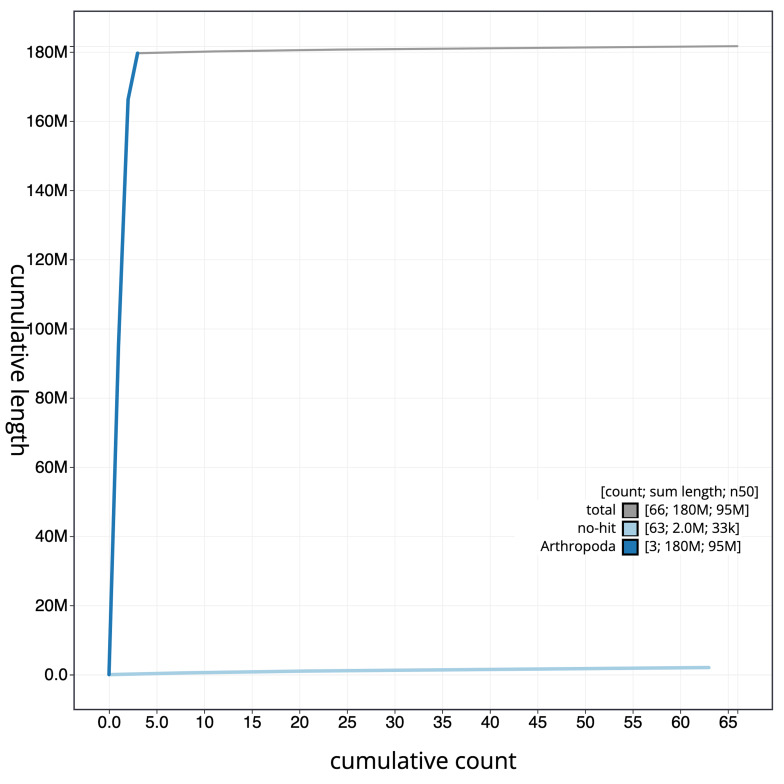
Cumulative chromosome length for *An. darlingi* assembly idAnoDarlMG_H_01. The grey line shows cumulative length for all chromosomes. Coloured lines show cumulative lengths of chromosomes assigned to each phylum using the buscogenes taxrule. The interactive version of this figure is available at https://blobtoolkit.genomehubs.org/view/idAnoDarlMG_H_01/dataset/CALSDZ01/cumulative.

**Figure 4.**
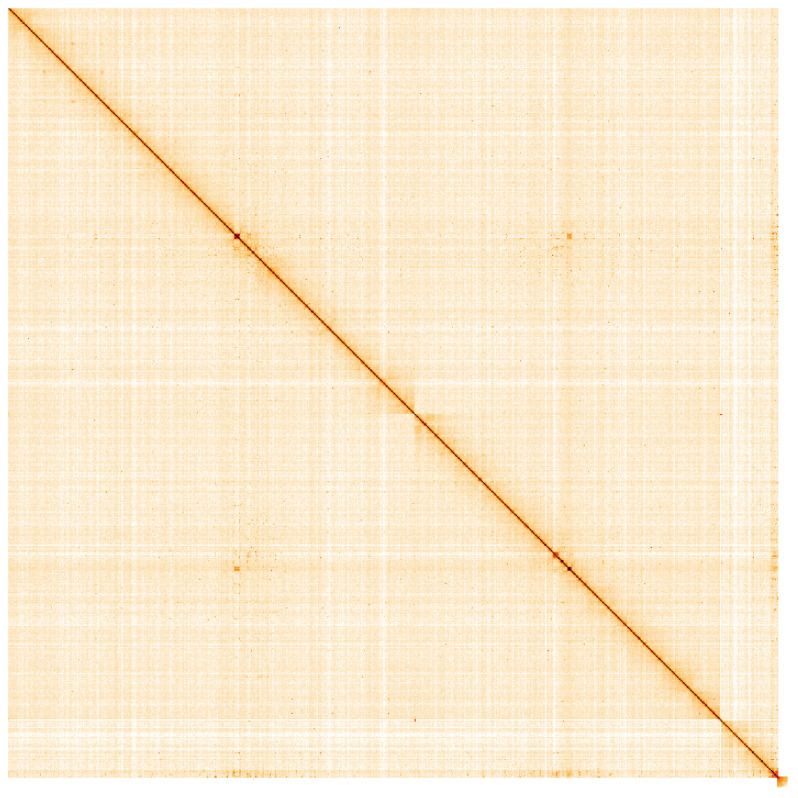
Genome assembly of *An. darlingi*, idAnoDarlMG_H_01: Hi-C contact map. Visualised in HiGlass. Chromosomes are arranged in size order from left to right and top to bottom. The interactive Hi-C map can be viewed at https://genome-note-higlass.tol.sanger.ac.uk/l/?d=Pap61sCkTXaWekXO6tp-Wg.

**Table 2.** Chromosomal pseudomolecules in the genome assembly of *An. darlingi* from French Guiana, idAnoDarlMG_H_01.

INSDC accession	Chromosome	Size (Mb)	Count	Gaps
**OX030911.1**	2RL	94.951	1	10
**OX030912.1**	3RL	71.271	1	5
**OX030913.1**	X	13.401	1	15
**OX030914.1**	MT	0.015	1	0
	2 Unlocalised	0.030	1	0
	X Unlocalised	1.957	60	0
	Unplaced	0.026	1	0

The assembly has a BUSCO 5.3.2 ^9^ completeness of 97.2% using the diptera_odb10 reference set. While not fully phased, the assembly deposited is of one haplotype. Contigs corresponding to the second haplotype have also been deposited.

## Genome sequence report for specimen from Peru

The genome was sequenced from a single female *Anopheles darlingi* from an established mosquito colony that originated from Cahuide, Peru (–4.230, –73.460). Unfortunately, this colony no longer exists and is therefore not available for further research. A total of 68-fold coverage in Pacific Biosciences single-molecule HiFi long reads (N50 10.925 kb) and 113-fold coverage in 10X Genomics read clouds were generated. Primary assembly contigs were scaffolded with chromosome conformation Hi-C data from a female sibling. Manual assembly curation corrected 26 missing joins or misjoins and removed 1 duplicated haplotig.

**Figure 5.**
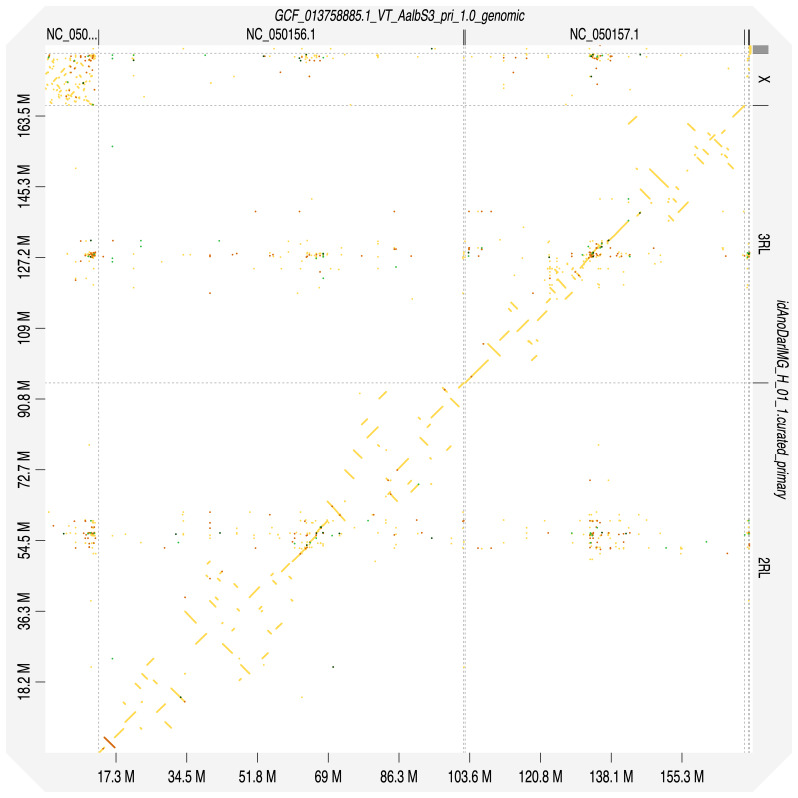
Synteny between genome assemblies of *An. albimanus* AalbS3 and *An. darlingi*, idAnoDarlMG_H_01. Chromosome arms arrangements are identical, while within arms there are numerous rearrangements.

**Figure 6.**
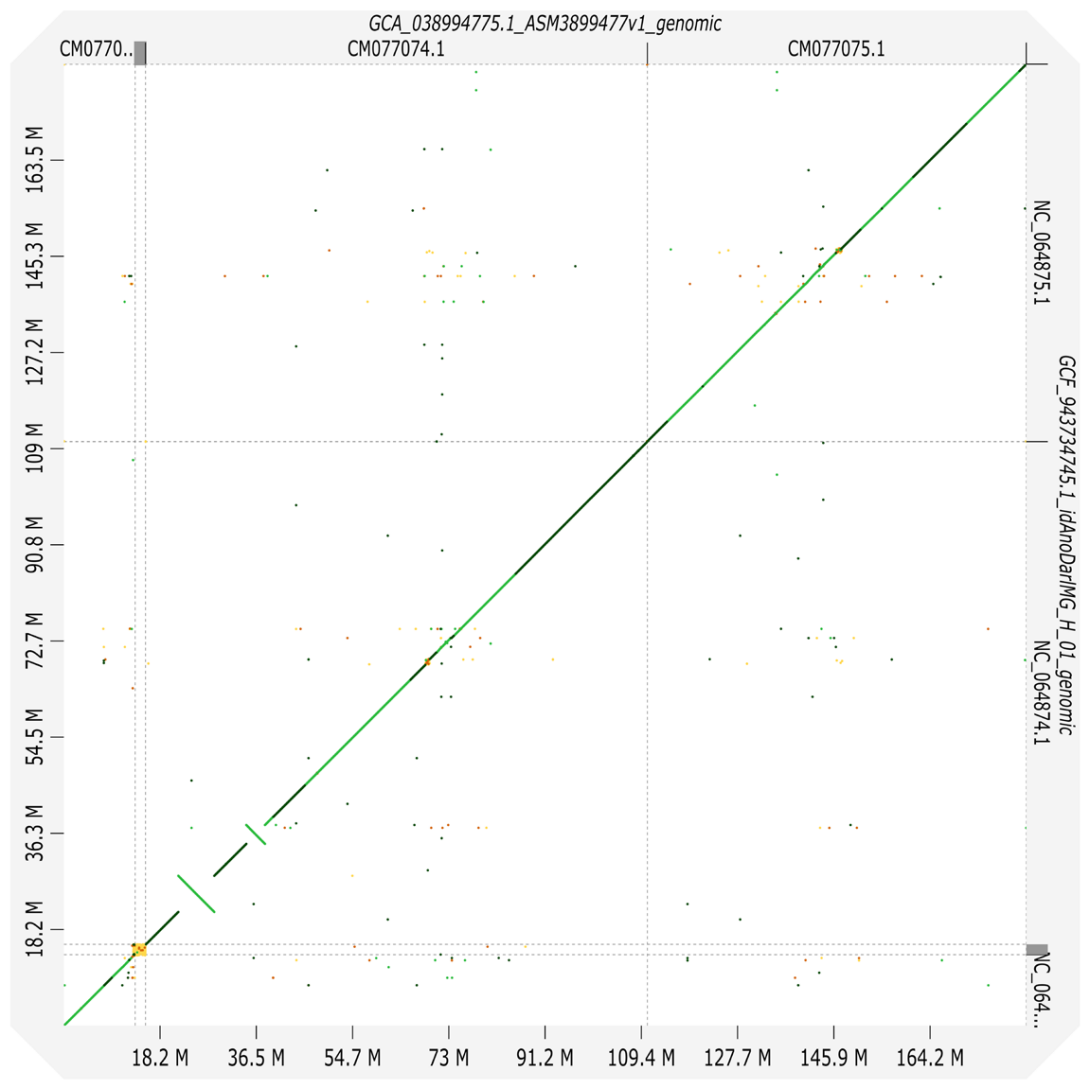
Synteny between genome assemblies of *An. darlingi* from French Guiana, idAnoDarlMG_H_01, and from Peru, idAnoDarlJC-H15_27. Coordinates of two inversions are provided in Table 5.

The final assembly has a total length of 182 Mb in 54 sequence scaffolds with a scaffold N50 of 95.091 Mb ( Table 3). 98.92% of the assembly sequence was assigned to three chromosomal-level scaffolds, representing two autosomes and the X sex chromosome ( Table 4). Synteny between this assembly and that of *An. albimanus* AalbS3 showed identical chromosome arms, with numerous rearrangements within the arms ( Figure 5). Compared to the assembly generated from a female from French Guiana, this assembly has two large scale inversions in chromosome arm 2R ( Figure 6; Table 5).

**Table 3.** Genome data for *Anopheles darlingi* from Peru, idAnoDarlJC-H15_27.

*Project accession data*	
Assembly identifier	ASM3899477v1
Species	*Anopheles darlingi*
Specimen	idAnoDarlJC-H15_27
NCBI taxonomy ID	43151
BioProject	PRJNA830873
BioSample ID	SAMN33940061
Isolate information	female, whole organism
*Raw data accessions*	
PacificBiosciences SEQUEL II	SRX26935726
10X Genomics Illumina	SRX26935727, SRX26935728, SRX26935729, SRX26935730
Hi-C Illumina	SRX26803935
*Genome assembly*	
Assembly accession	GCA_038994775
Span (Mb)	182.384
Number of contigs	86
Contig N50 length (Mb)	13.497
Number of scaffolds	54
Scaffold N50 length (Mb)	95.091
Longest scaffold (Mb)	95.091
BUSCO * genome score	C:97.0%[S:96.3%,D:0.7%], F:0.7%,M:2.3%,n:3285

* BUSCO scores based on the diptera_odb10 BUSCO set using BUSCO 5.3.2. C=complete [S=single copy, D=duplicated], F=fragmented, M=missing, n=number of orthologues in comparison.

**Table 4.** Chromosomal pseudomolecules in the genome assembly of *An. darlingi* from Peru, idAnoDarlJC-H15_27.

INSDC accession	Chromosome	Size (Mb)	Count
**CM077074.1**	2RL	95.091	1
**CM077075.1**	3RL	71.801	1
**CM077073.1**	X	13.508	1
**CM077077.1**	MT	0.015	1
	X Unlocalised	1.974	52

**Table 5.** Inversions between genome assemblies of *An. darlingi* from French Guiana and Peru.

INSDC accession	Chromosome	Start	End
**OX030911.1**	2RL	6,114,428	12,943,365
**OX030911.1**	3RL	18,970,078	21,986,145

The assembly has a BUSCO 5.3.2 ^9^ completeness of 97.0% using the diptera_odb10 reference set.

## Chromosome arms delineation

**Figure 7.**
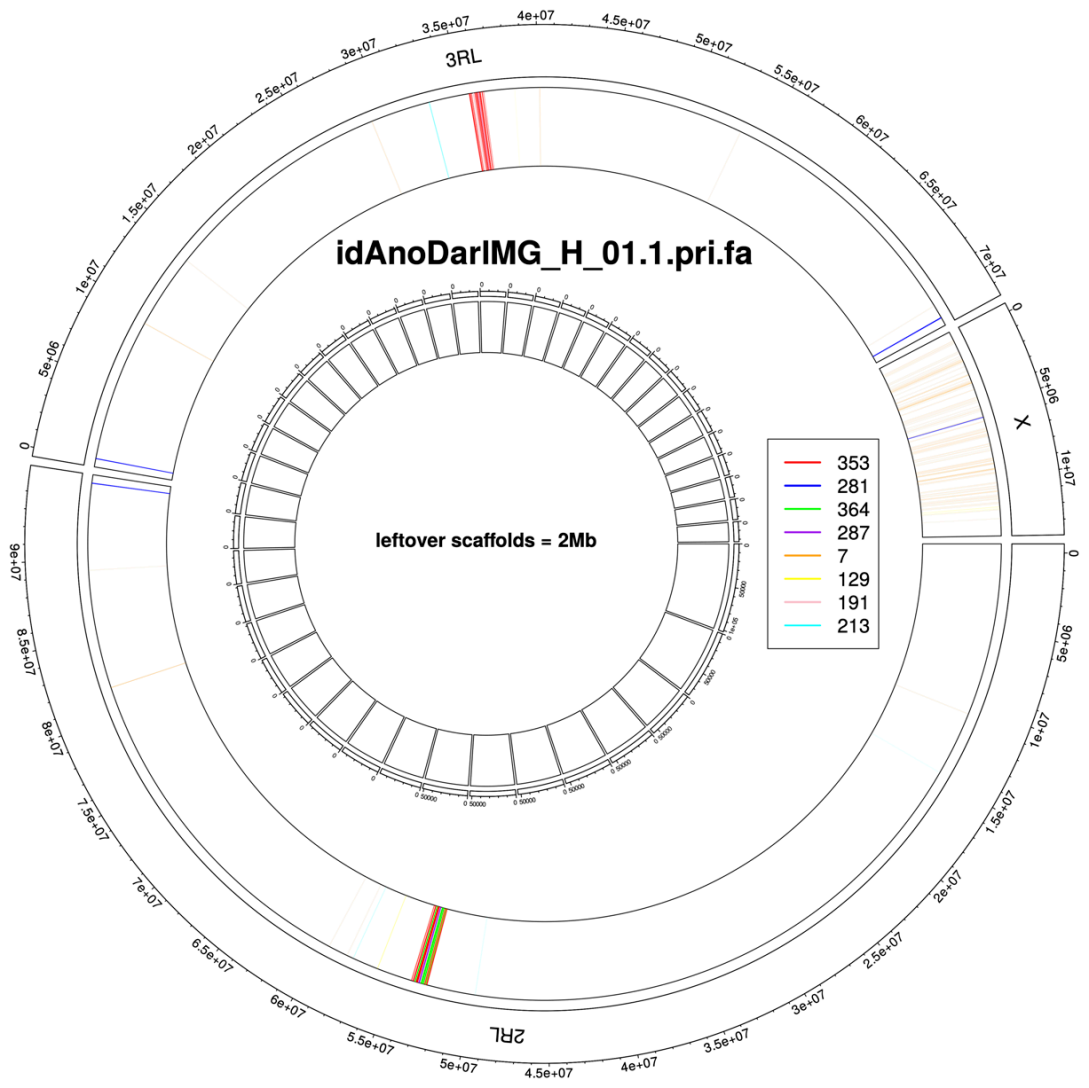
Tandem repeats annotation for genome assembly of *An. darlingi* from French Guiana, idAnoDarlMG_H_01.

**Figure 8.**
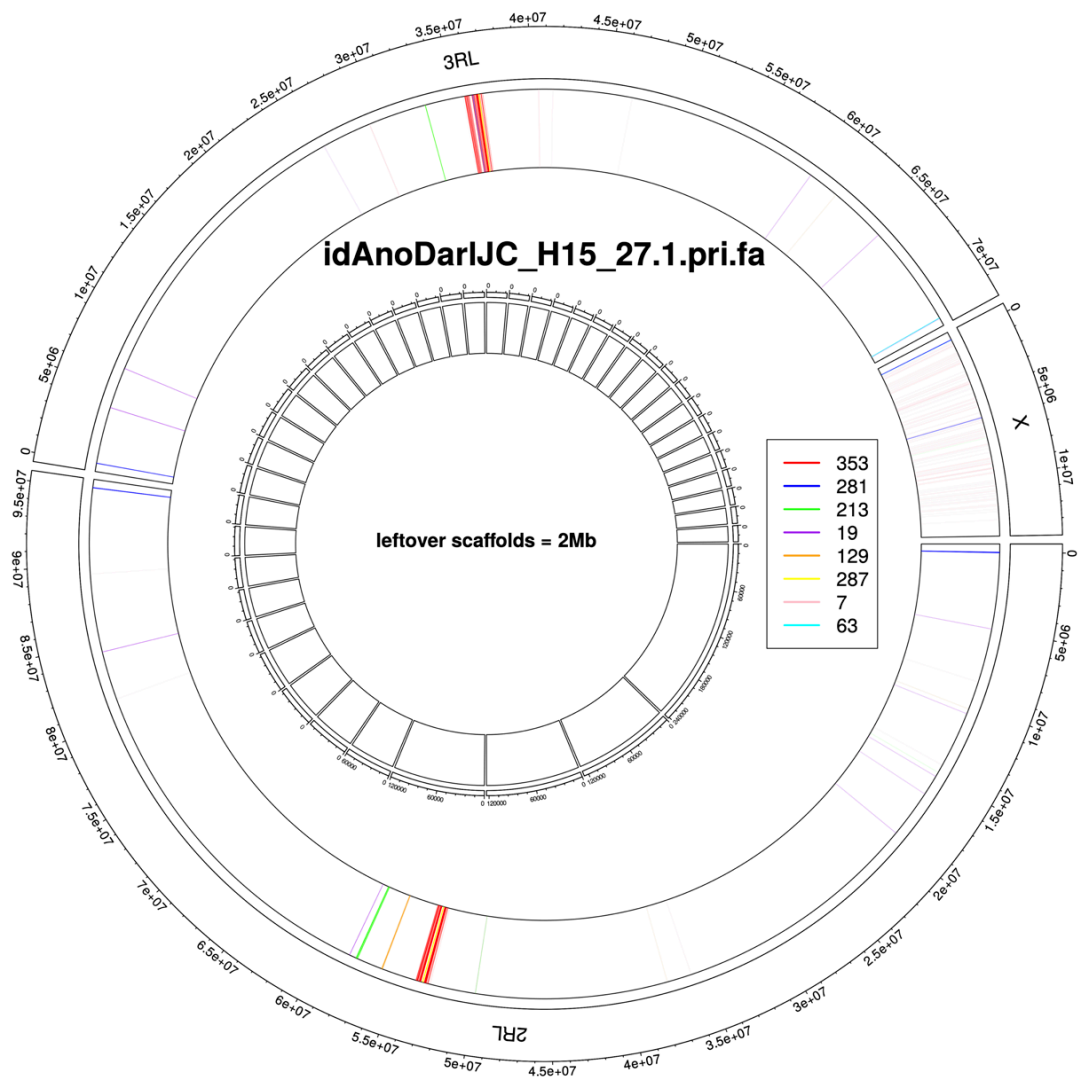
Tandem repeats annotation for genome assembly of *An. darlingi* from Peru, idAnoDarlJC-H15_27.

To facilitate the delineation of chromosome arms in both assemblies, we predicted most common tandem repeats using TRASH ^10^. In autosomes, largest blocks of tandem repeats coincided with expected centromere locations and were mostly composed of repeats with 353 base long monomers ( Figure 7, Figure 8). We used outermost copies of this repeat to annotate chromosome arms ( Table 6). In the X chromosome, no large tandem repeat blocks were observed on the centromere adjacent side, i.e. at the end of the chromosome sequence. In both assemblies, the majority of chromosome arms ended with tandem repeats with 281 base long monomers, suggesting that these repeats act as telomeres – non-canonical satellite repeats are characteristic for other *Anopheles* species ^8^.

**Table 6.** Chromosome arms annotation in genome assemblies of *An. darlingi*.

Chromosome arm	idAnoDarlMG_H_01 start	idAnoDarlMG_H_01 end	idAnoDarlJC-H15_27 start	idAnoDarlJC-H15_27 end
**2R**	1	52,837,268	1	52,904,211
**2L**	53,277,590	94,951,917	53,898,552	95,090,923
**3R**	1	35,602,462	1	35,719,087
**3L**	36,556,652	71,270,736	36,907,605	71,801,356

## Methods

### Sample acquisition and nucleic acid extraction

*Anopheles darlingi* offspring were reared from a wild caught gravid female collected from Macouria, French Guiana (5.015, –52.528) by Katy Heu and Romuald Carinci in April 2019. Individuals from the resulting brood were shipped on dry ice to the UK. From this brood, a single female idAnoDarlMG-H_01 was used for Pacific BioSciences and its sibling female idAnoDarlMG-H_08 was used for Arima Hi-C.

*Anopheles darlingi* offspring from Peru were reared from a colony in April 2019. The original collection to create this colony used wild females collected from Cahuide, Loreto, Peru (–4.230, –73.460) by the Amazonian International Center of Excellence in Malaria Research (Amazonian ICEMR) team in a long-term project led by Dionicia Gamboa, Marta Moreno, Jan Conn and Joseph Vinetz ^6^. A single mated female from this colony was used to rear a brood to be used for sequencing. From this brood, female sibling idAnoDarlJC-H15_27 was used for Pacific BioSciences and 10x linked reads, and female sibling idAnoDarlJC-H15_22 was used for Arima Hi-C.

For both reference genomes, high molecular weight (HMW) DNA extraction used one whole insect disrupted by manual grinding with a blue plastic pestle in Qiagen MagAttract lysis buffer and then extracted using the Qiagen MagAttract HMW DNA extraction kit with two minor modifications ^11^. The quality of the DNA was evaluated using an Agilent FemtoPulse to ensure that most DNA molecules were larger than 30 kb, and preferably > 100 kb. In general, single mosquito extractions range in total estimated DNA yield from 200 ng to 800 ng, with an average yield of 500 ng. Low molecular weight DNA was removed using a 0.8X AMpure XP purification. For the specimen from Peru, a small aliquot (less than ~5% of the total volume) of HMW DNA was set aside for 10X Linked Read sequencing. For PacBio sequencing, DNA was sheared to an average fragment size of 12–20 Kb using a Diagenode Megaruptor 3 at speeds ranging from 27 to 30. Sheared DNA was purified using AMPure PB beads with a 1.8X ratio of beads to sample to remove the shorter fragments and concentrate the DNA sample. The concentration and quality of the sheared and purified DNA was assessed using a Nanodrop spectrophotometer and Qubit Fluorometer with the Qubit dsDNA High Sensitivity Assay kit. Fragment size distribution was evaluated by running the sheared and cleaned sample on the FemtoPulse system once more. The median DNA fragment size for *Anopheles* mosquitoes was 15 kb and the median yield of sheared DNA was 200 ng, with samples typically losing about 50% of the original estimated DNA quantity through the process of shearing and purification.

For Hi-C data generation, separate sibling mosquito specimens were used as input material for the Arima V2 Kit according to the manufacturer’s instructions for animal tissue. This approach of using a sibling was taken in order to enable all material from a single specimen to contribute to the PacBio data generation given we were not always able to meet the minimum suggested guidance of starting with > 300 ng of HMW DNA from a specimen. Samples proceeded to the Illumina library prep stage even if they were suboptimal (too little tissue) going into the Arima reaction.

To assist with annotation for assembly of specimen from French Guiana, generated by NCBI RefSeq ^12^ and available through VectorBase ^13^ under accession AdarGF1, RNA was extracted from separate whole sibling and unrelated insect specimens (idAnoDarlMG-G_01, idAnoDarlMG-G_02, and idAnoDarlMG-H_11) using TRIzol, according to the manufacturer’s instructions. RNA was then eluted in 50 μl RNAse-free water, and its concentration was assessed using a Nanodrop spectrophotometer and Qubit Fluorometer using the Qubit RNA Broad-Range (BR) Assay kit. Analysis of the integrity of the RNA was done using Agilent RNA 6000 Pico Kit and Eukaryotic Total RNA assay. Samples were not always ideally preserved for RNA, so qualities varied but all were sequenced anyway.

### Sequencing

We prepared libraries as per the PacBio procedure and checklist for SMRTbell Libraries using Express TPK 2.0 with low DNA input. Every library was barcoded to support multiplexing. Final library yields ranged from 20 ng to 100 ng, representing only about 25% of the input sheared DNA. Libraries from two specimens were typically multiplexed on a single 8M SMRT Cell. Sequencing complexes were made using Sequencing Primer v4 and DNA Polymerase v2.0. Sequencing was carried out on the Sequel II system with 24-hour run time and 2-hour pre-extension. A 10X Genomics Chromium read cloud sequencing library was also constructed according to the manufacturer’s instructions (this product is no longer available). Only 0.5 ng of DNA was used and only 25–50% of the gel emulsion was put forward for library prep due to the small genome size. For Hi-C data generation, following the Arima HiC V2 reaction, samples were processed through Library Preparation using a NEB Next Ultra II DNA Library Prep Kit and sequenced aiming for 100x depth. RNA libraries were created using the directional NEB Ultra II stranded kit. Sequencing was performed by the Scientific Operations core at the Wellcome Sanger Institute on Pacific Biosciences SEQUEL II (HiFi), Illumina NovaSeq 6000 (10X and Hi-C), or Illumina HiSeq 4000 (RNAseq).

### Genome assembly

Assemblies were carried out with Hifiasm ^14^; haplotypic duplications were identified and removed with purge_dups ^15^. For the specimen from Peru, one round of polishing was performed by aligning 10X Genomics read data to the assembly with Long Ranger ALIGN, calling variants with FreeBayes ^16^. The assembly was then scaffolded with Hi-C data ^17^ using SALSA2 ^18^. The assembly was checked for contamination as described previously ^19^. Manual curation was performed using gEVAL ^20^, HiGlass ^21^ and Pretext ^22^. The mitochondrial genome was assembled using MitoHiFi ^23^, which performs annotation using MitoFinder ^24^. The genome was analysed and BUSCO scores were generated within the BlobToolKit environment ^25^. Synteny analyses were performed with D-GENIES ^26^. Tandem repeats were annotated with TRASH ^10^. Table 7 contains a list of all software tool versions used, where appropriate.

**Table 7.** Software tools used.

Software tool	Version	Source
hifiasm	0.14	14
purge_dups	1.2.3	15
longranger align	2.2.2	27
freebayes	1.3.1	16
SALSA2	2.2-4c80ac1	18
MitoHiFi	2	23
gEVAL	N/A	20
HiGlass	1.11.6	21
PretextView	0.1.x	22
BlobToolKit	3.4.0	28
BUSCO	5.3.2	9
D-GENIES	1.4	26
TRASH	1.2	10

## Ethics/compliance issues

The genetic resources accessed and utilised under this project were done so in accordance with the UK ABS legislation (Nagoya Protocol (Compliance) (Amendment) (EU Exit) Regulations 2018 (SI 2018/1393)) and the national ABS legislation within the country of origin, where applicable.

## Data Availability

European Nucleotide Archive: PRJEB53253, https://identifiers.org/bioproject/PRJEB53253; PRJNA830873, https://identifiers.org/bioproject/PRJNA830873. The genome sequences are released openly for reuse. The *Anopheles darlingi* genome sequencing initiative is part of the Anopheles Reference Genomes project PRJEB51690. All raw sequence data and the assemblies have been deposited in INSDC databases. Raw data and assembly accession identifiers are reported in Table 1 and Table 3.

## Author information

Members of the Wellcome Sanger Institute Scientific Operations: Sequencing Operations collective are listed here: https://doi.org/10.5281/zenodo.4790456.
