# Supplementary material for: Two chromosomal reference genome sequences for the malaria mosquito, Anopheles ( Nyssorhynchus) darlingi, Root, 1926 from French Guiana and Peru
Source: Wellcome Open Res. 2025 Apr 10;10:187. [Version 1] doi: 10.12688/wellcomeopenres.23989.1 (PMC12485361; doi:10.12688/wellcomeopenres.23989.1)
Supplement: Peer review history

# Reviewer report: Reviewer response for version 1

**DOI:** 10.21956/wellcomeopenres.26465.r122270

**Published:** 2025-05-10

**Authors:** Komal Kumar Bollepogu Raja

**Affiliations:** 1 Baylor College of Medicine, Houston, USA

The manuscript by Gendrin et al presents two high quality genome assembly sequences for the species *Anopheles* *darlingi* from French Guiana and Peru. The authors have achieved 182 Mb of assemblies with N50 of about 95 Mb and BUSCO completeness of >97% suggesting good quality of the genome sequences. This assembly is a substantial improvement over the fragmented 2013 version of the genome. The authors have sufficiently introduced the biology and evolutionary context of the species including colony origin. The experimental and bioinformatics methodology is robust. The use of TRASH for centromeric and teloomeric repeat annotation is commendable.  Overall, this manuscript represents a significant advance in *An. darlingi* genomics and will be a valuable resource for malaria vector research.

**Minor comment**

 Please be consistent between 'idAnoDarlMG-H_01' and 'idAnoDarlMG_H_01'

Are sufficient details of methods and materials provided to allow replication by others?

Yes

Is the rationale for creating the dataset(s) clearly described?

Yes

Are the datasets clearly presented in a useable and accessible format?

Yes

Are the protocols appropriate and is the work technically sound?

Yes

Reviewer Expertise:

Single cell genomics, bioinformatics, genomics and Neurobiology

I confirm that I have read this submission and believe that I have an appropriate level of expertise to confirm that it is of an acceptable scientific standard.

# Reviewer report: Reviewer response for version 1

**DOI:** 10.21956/wellcomeopenres.26465.r122268

**Published:** 2025-05-10

**Authors:** Renee Ali

**Affiliations:** 1 Molecular Microbiology and Immunology, Johns Hopkins University Bloomberg School of Public Health, Baltimore, Maryland, USA

A good addition to the genomic data available for this important neotropical vector *Anopheles darlingi*. The methods were well explained and reproducible. It would be nice to produce another assembly or compare to the first version if possible to observe whether there where also inversions on the chromosome arm 2R.

Are sufficient details of methods and materials provided to allow replication by others?

Yes

Is the rationale for creating the dataset(s) clearly described?

Yes

Are the datasets clearly presented in a useable and accessible format?

Yes

Are the protocols appropriate and is the work technically sound?

Yes

Reviewer Expertise:

Genetics, mosquito populations, understudied vectors

I confirm that I have read this submission and believe that I have an appropriate level of expertise to confirm that it is of an acceptable scientific standard.

# Reviewer report: Reviewer response for version 1

**DOI:** 10.21956/wellcomeopenres.26465.r122272

**Published:** 2025-04-26

**Authors:** Craig Wilding

**Affiliations:** 1 University College Dublin School of Biology and Environmental Science (Ringgold ID: 123708), Dublin, Leinster, Ireland

In this article, the authors describe the generation and annotation of chromosomal-scale genome sequences from two separate *Anopheles darlingi* individuals collected from geographically isolated (French Guiana and Peru) populations. Both genomes are similarly high quality (in terms of scaffold-length and BUSCO completeness) and were generated using a combination of PacBio HiFi and Hi-C scaffolding for genome sequencing, and Illumina for RNASeq (on a specimen from French Guiana).

 Having two assemblies has permitted the authors to identify two major chromosomal inversions on chromosome 2R.

 The methods utilized are reported clearly although syntenic comparison for the French Guianan sample was reported to be undertaken versus the “ *An. albimanus* STECLA strain assembly (GCA_013758885.1)” whilst synteny for the Peruvian sample is reported as generated against “ *An. albimanus *AalbS3”. Whilst I think these are the same, for clarity, it would be better to be consistent in reference to the *An. albimanus* strain. Note, I believe that it should be the STELCA strain (not STECLA).

Are sufficient details of methods and materials provided to allow replication by others?

Yes

Is the rationale for creating the dataset(s) clearly described?

Yes

Are the datasets clearly presented in a useable and accessible format?

Yes

Are the protocols appropriate and is the work technically sound?

Yes

Reviewer Expertise:

Evolutionary genetics/genomics

I confirm that I have read this submission and believe that I have an appropriate level of expertise to confirm that it is of an acceptable scientific standard.
